# Supplementary material for: The AhR Is a Critical Regulator of the Pulmonary Response to Cannabis Smoke
Source: FASEB J. 2025 Sep 17;39(18):e71027. doi: 10.1096/fj.202501533R (PMC12442951; doi:10.1096/fj.202501533R)
Supplement: Supplementary file 1 — Data S1: Supporting Information. [file FSB2-39-e71027-s001.zip › 202501533R-sup-0003-SI_Figure-S01.pdf]

Online Supplement

The AhR is a Critical Regulator of the Pulmonary Response to Cannabis Smoke

Emily T. Wilson[1,2], Roham Gorgani[2,3], Nicole Heimbach[1,2], Alexandra Bartolomucci[2,3], Thupten Tsering[2,3], Julia V. Burnier[2,3,4], David H. Eidelman[2,5], and Carolyn J. Baglole[1,2,3,5*]

[1]Department of Pharmacology and Therapeutics, McGill University, Montreal, Quebec Canada
[2]Research Institute of the McGill University Health Centre, Montreal, Quebec Canada
[3]Department of Pathology, McGill University, Montreal, Quebec Canada
[4]Gerald Bronfman Department of Oncology, McGill University, Montreal, Canada
[5]Department of Medicine, McGill University, Montreal, Canada

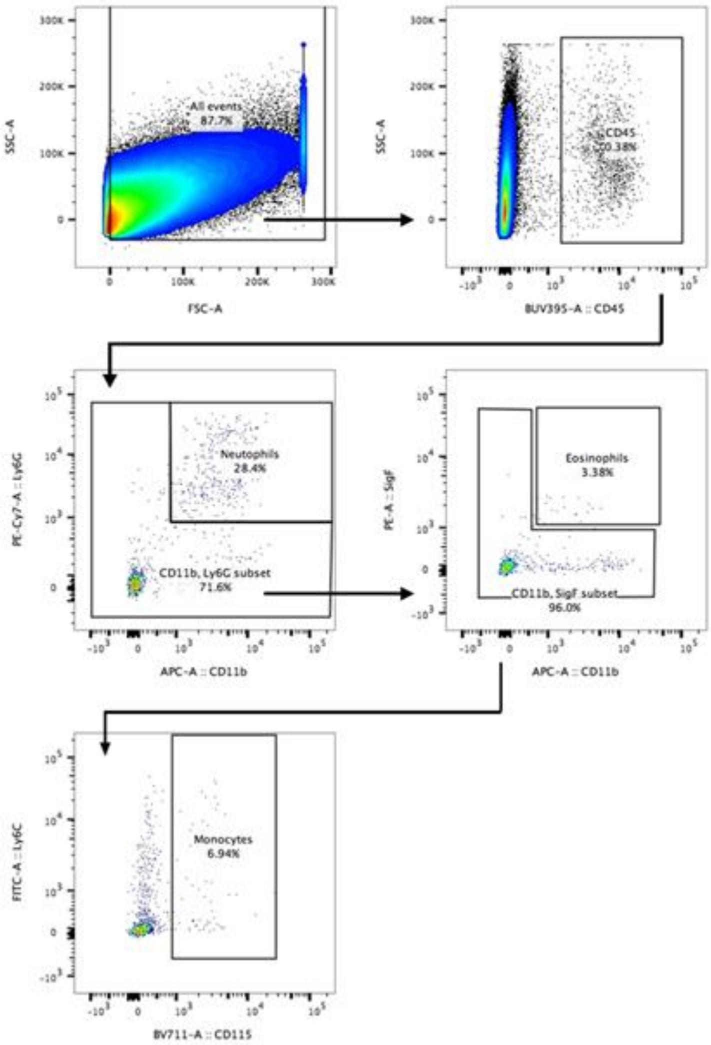

Supp. Figure 1 Gating Strategy for Innate Immune Cells in the Blood Total events were selected using forward scatter (FSC-A) and side scatter (SSC-A) parameters. $CD45^+$ leukocytes were gated to exclude non-immune cells. Among $CD45^+$ cells, neutrophils were identified as $CD11b^+Ly6G^+$ cells, while the $CD11b^+Ly6G^-$ subset was further analyzed for eosinophils and monocytes. Eosinophils were gated based on SiglecF (SigF) expression, and monocytes were identified as $CD115^+Ly6C^+$ cells.

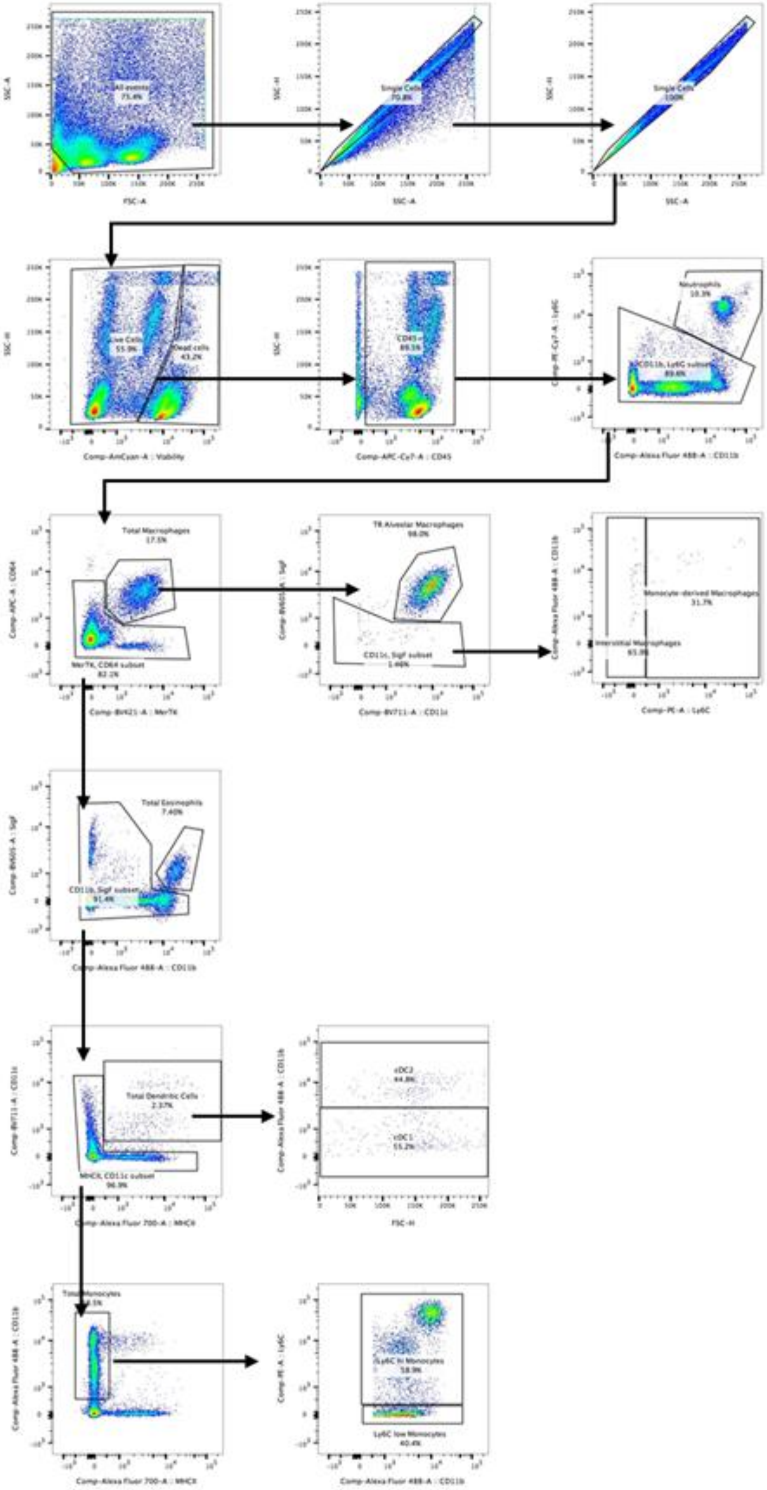

Supp. Figure 2 Gating Strategy for Innate Immune Cells of the Lung Tissue Total events were selected using forward scatter (FSC-A) and side scatter (SSC-A) parameters to remove debris. Single cells were identified using SSC-A vs. SSC-H and FSC-A vs. FSC-H plots. Viable $CD45^{+}$ immune cells were gated to exclude dead cells and non-immune populations.

Among $CD45^+$ cells, neutrophils were identified as $CD11b^+Ly6G^+$, while the negative subset was further analyzed for macrophages, eosinophils, dendritic cells, and monocytes. Macrophages were classified into total macrophages ($MerTK^+CD64^+$), tissue-resident (TR) alveolar macrophages ($CD11c^+SiglecF^+$), monocyte-derived macrophages ($CD11c^-SiglecF^-Ly6C^+$), and interstitial macrophages ($CD11c^-SiglecF^-Ly6C^-$). Eosinophils were identified as $MerTK^-CD64^-SigF^+CD11b^+$. DCs were classified as $MHCII^+CD11c^+$, further subdivided into type 1 conventional DCs (cDC1; $CD11b^+$) and type 2 conventional DCs (cDC1; $CD11b^-$). Monocytes were subdivided into $Ly6C^{hi}$ inflammatory monocytes and $Ly6C^{low}$ patrolling monocytes.

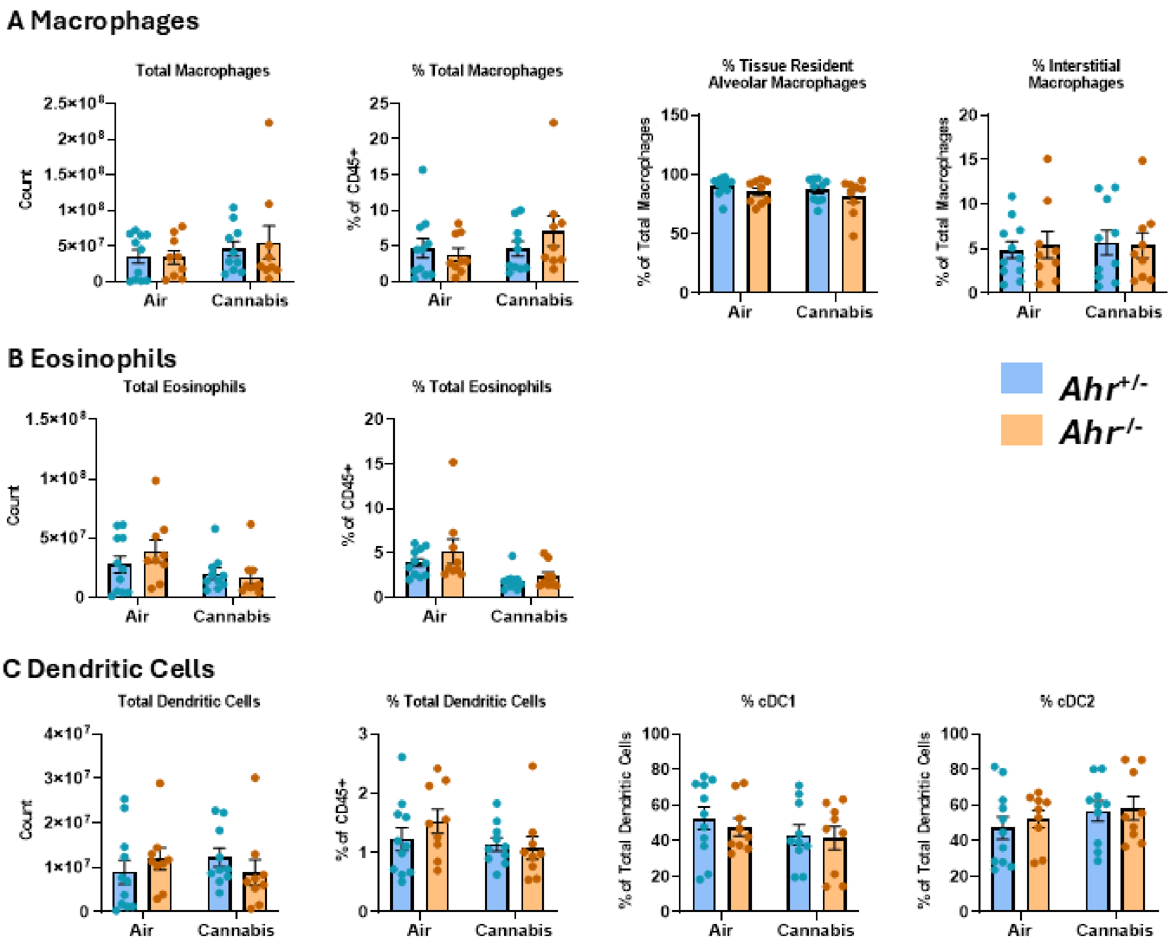

Supp. Figure 3 Quantification of Innate Immune Cell Populations in the Lung

(A) Macrophage populations, including total macrophage counts, percentage of total macrophages among $CD45^+$ cells, percentage of total macrophages that are tissue-resident alveolar macrophages, and percentage of total macrophages that are interstitial macrophages. (B) Eosinophil populations, including total eosinophil counts and percentage of $CD45^+$ cells. (C) Dendritic cell populations, including total dendritic cell counts, percentage of $CD45^+$ cells, and percentage of total dendritic cells that are cDC1 or cDC2. Statistical significance is shown as $*p < 0.05$, $**p < 0.01$, $***p < 0.001$, and $****p < 0.0001$.

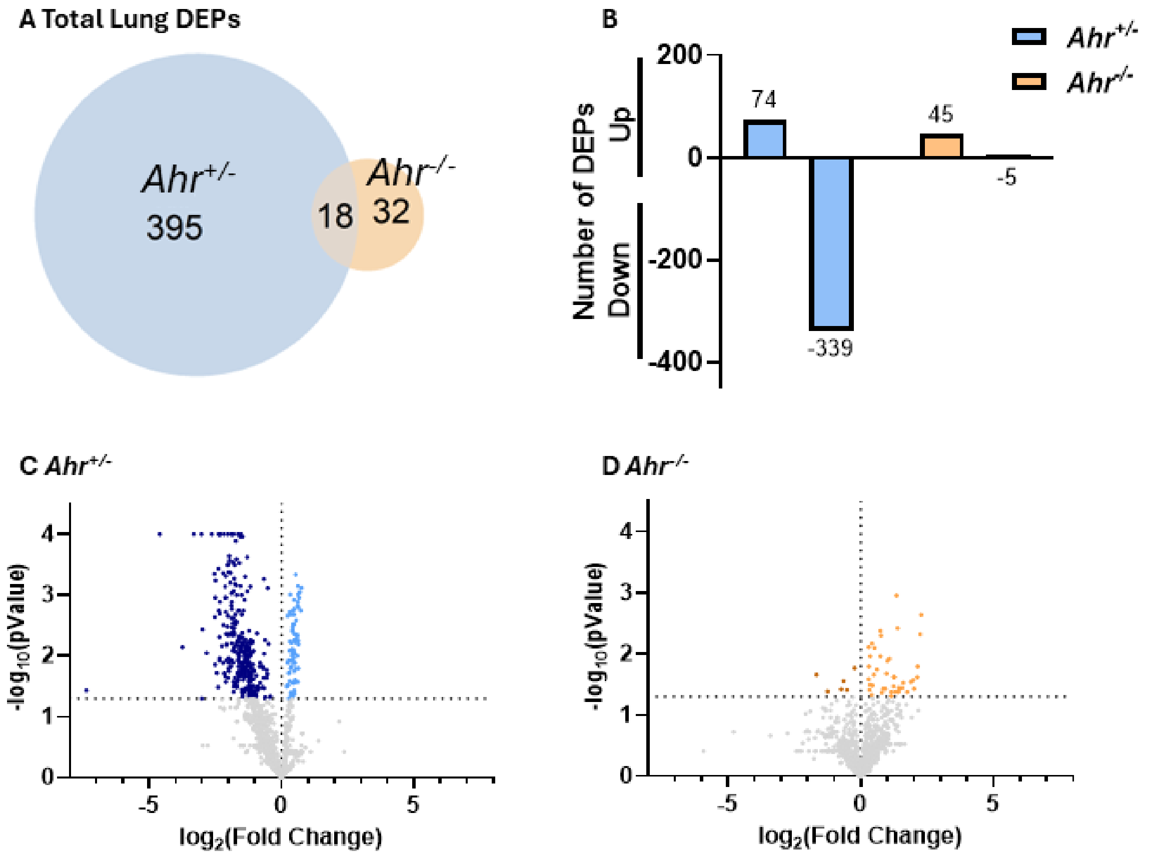

Supp. Figure 4 Lung Tissue DEPs in *Ahr*$^{+/-}$ and *Ahr*$^{-/-}$ Mice Exposed to Cannabis Smoke vs Air

(A) Venn diagram displaying the total number of DEPs in *Ahr*$^{+/-}$ and *Ahr*$^{-/-}$ mice exposed to cannabis smoke compared to air-exposed controls. (B) Bar graph quantifying the number of DEPs that are upregulated or downregulated in each genotype. (C, D) Volcano plots illustrating the distribution of DEPs in (C) *Ahr*$^{+/-}$ and (D) *Ahr*$^{-/-}$ mice. The horizontal dotted line indicates the statistical significance threshold (p<0.05).

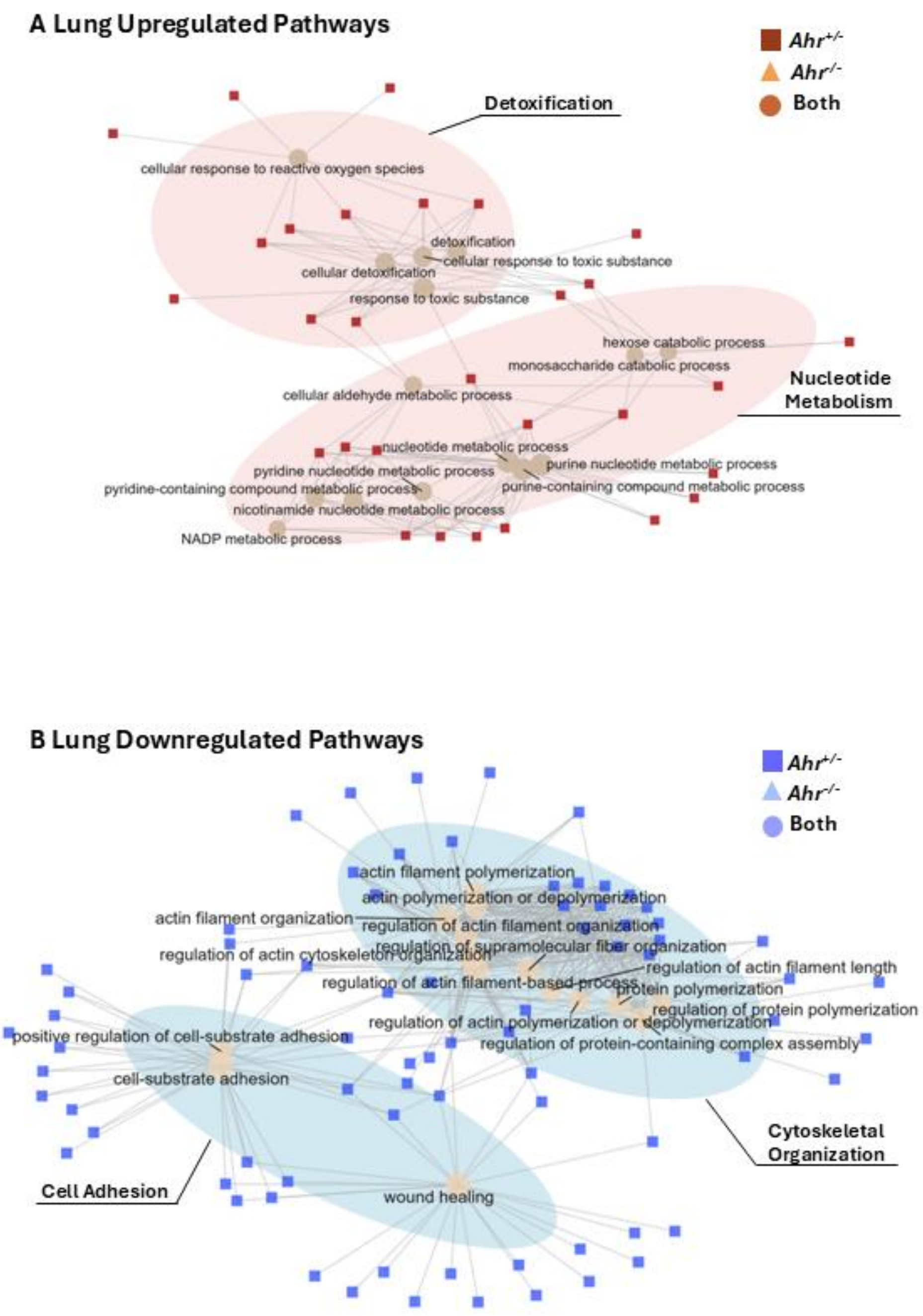

Supp. Figure 5 Labelled Network Analysis of Top 15 Upregulated and Downregulated Pathways in Lung Tissue

(A) Network analysis of the top 15 upregulated pathways in lung tissue following cannabis smoke exposure. (B) Network analysis of the top 15 downregulated pathways in lung tissue following cannabis smoke exposure. Beige pathway nodes are labeled with the GO term they represent, while the protein nodes are color-coded based on the genotype in which they are differentially expressed.

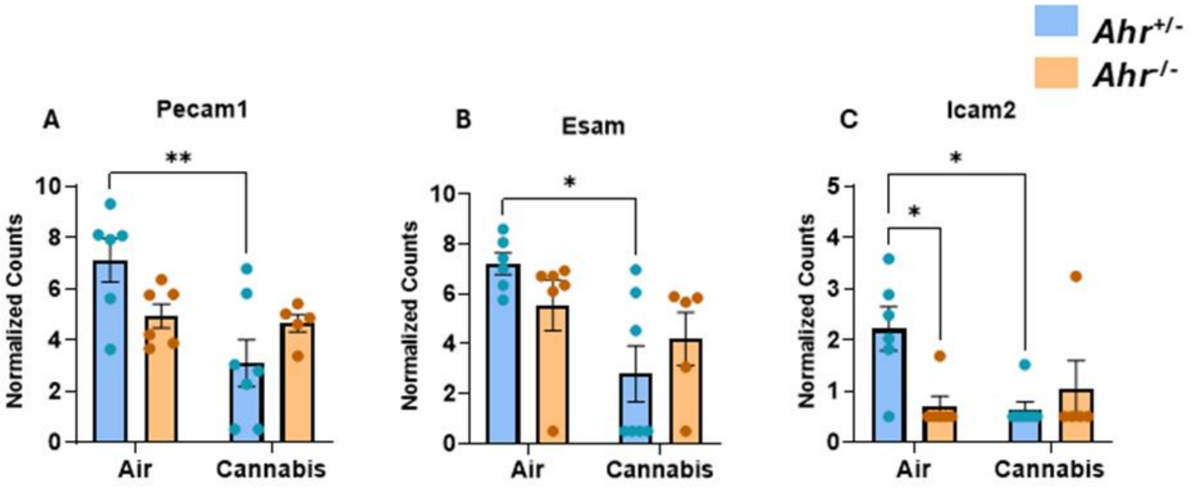

Supp. Figure 6 Levels Adhesion Molecules involved in Diapedesis in Lung Tissue of *Ahr*$^{+/-}$ and *Ahr*$^{-/-}$ Mice Exposed to Air or Cannabis Smoke.

Normalized counts of (A) Pecam1, (B) Esam, and (C) Icam2 in lung tissue. Data are presented as mean ± SEM, with individual data points shown. Statistical significance is denoted by $*p < 0.05$, and $**p < 0.01$.

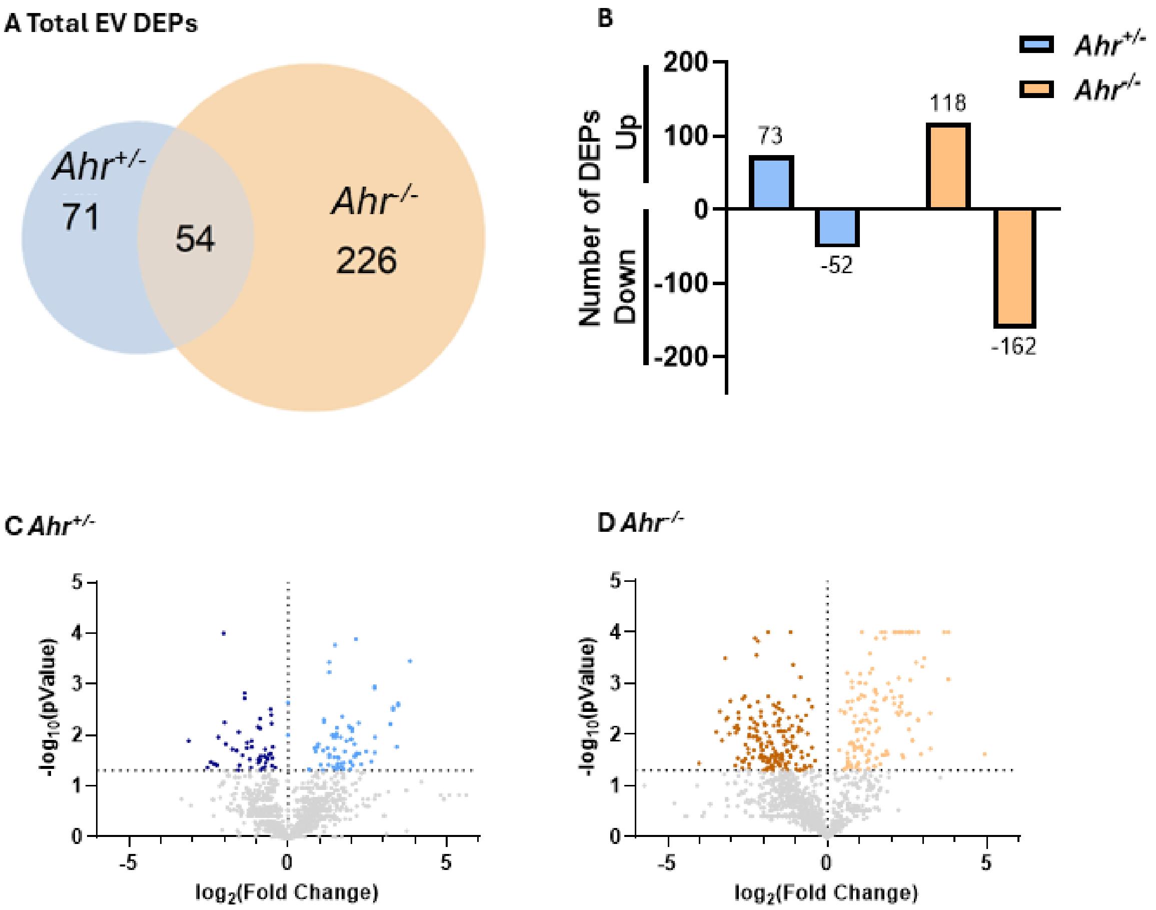

Supp. Figure 7 EV DEPs in *Ahr*$^{+/-}$ and *Ahr*$^{-/-}$ Mice Exposed to Cannabis Smoke vs Air

(A) Venn diagram displaying the total number of DEPs in *Ahr*$^{+/-}$ and *Ahr*$^{-/-}$ mice exposed to cannabis smoke compared to air-exposed controls. (B) Bar graph quantifying the number of DEPs that are upregulated or downregulated in each genotype. (C, D) Volcano plots illustrating the distribution of DEPs in (C) *Ahr*$^{+/-}$ and (D) *Ahr*$^{-/-}$ mice. The horizontal dotted line indicates the statistical significance threshold (p<0.05).

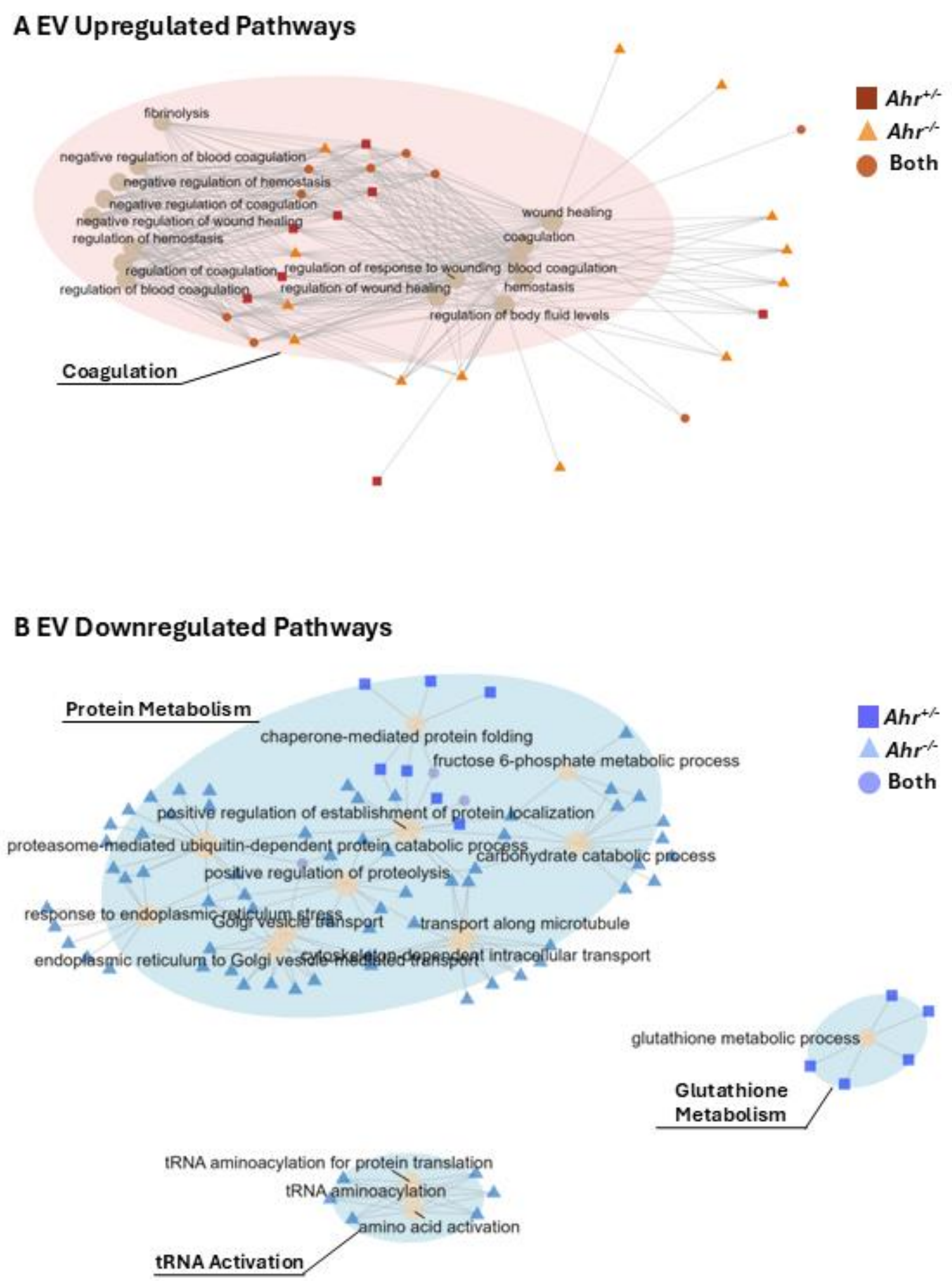

Supp. Figure 8 Labelled Network Analysis of Top 15 Upregulated and Downregulated Pathways in EVs.

(A) Network analysis of the top 15 upregulated pathways in EVs following cannabis smoke exposure. (B) Network analysis of the top 15 downregulated pathways in EVs following cannabis smoke exposure. Beige pathway nodes are labeled with the GO term they represent, while the protein nodes are color-coded based on the genotype in which they are differentially expressed.

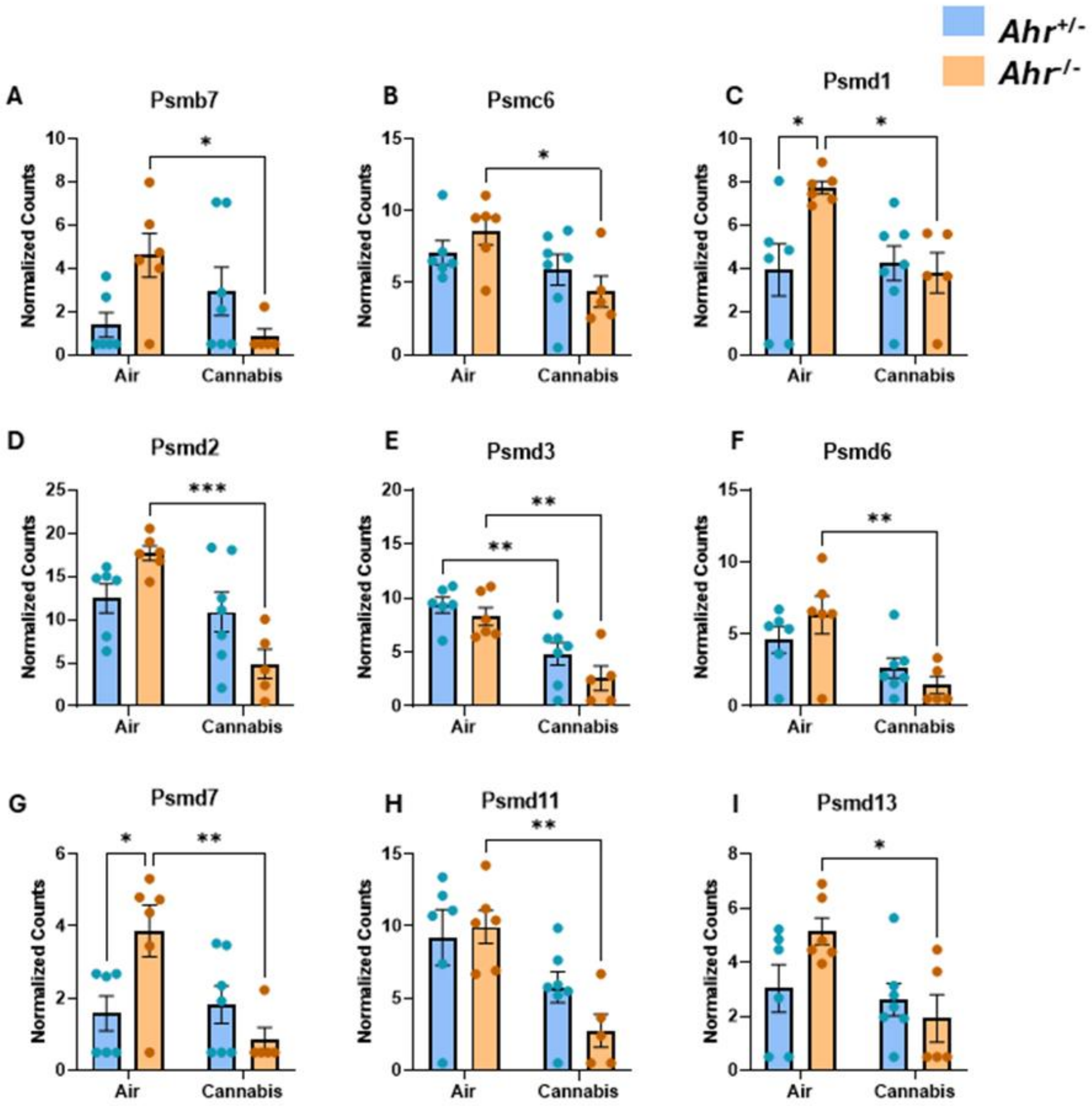

Supp. Figure 9 Proteasome Subunits in EVs From *Ahr*$^{+/-}$ and *Ahr*$^{-/-}$ Mice Exposed to Either Air or Cannabis Smoke.

Normalized counts for (A) Psmb7, (B) Psmc6, (C) Psmd1, (D) Psmd2, (E) Psmd3, (F) Psmd6, (G) Psmd7, (H) Psmd11, and (I) Psmd13. Data are presented as mean ± SEM, with individual data points shown. Statistical significance is denoted by *$p < 0.05$, **$p < 0.01$, and ***$p < 0.001$.

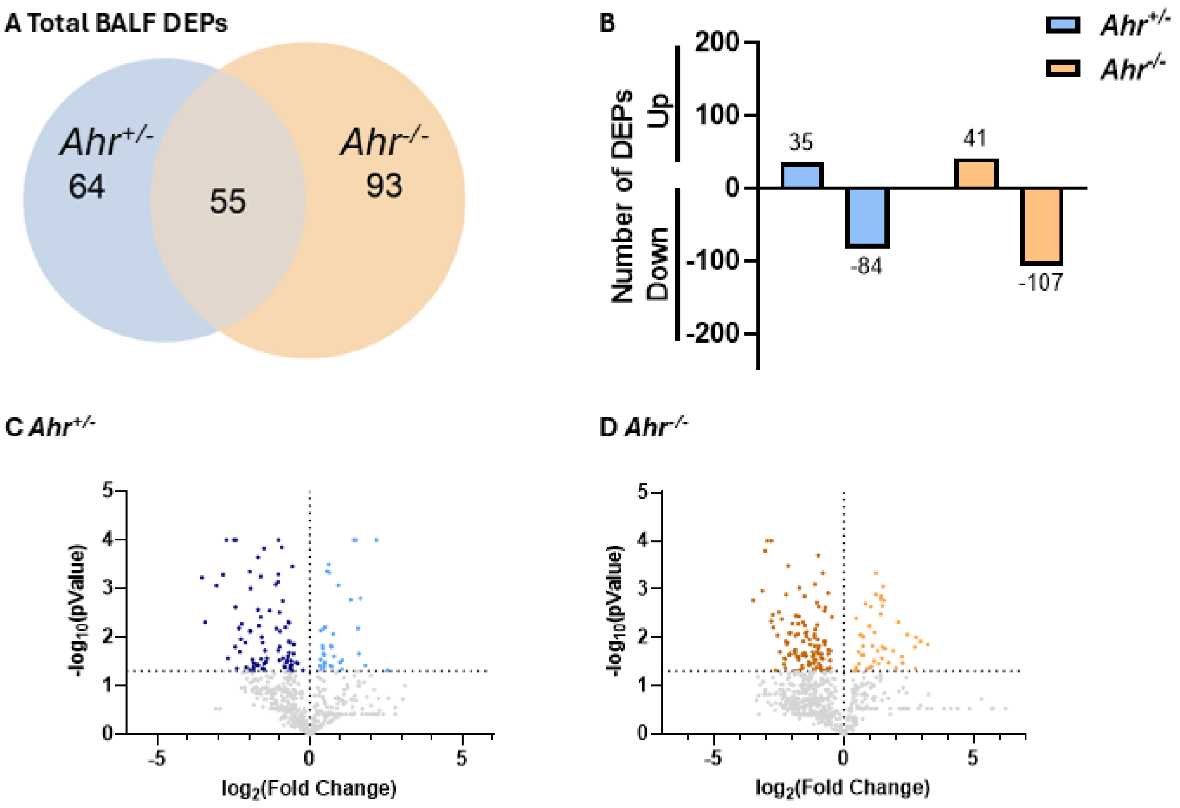

Supp. Figure 10 BALF DEPs in *Ahr*$^{+/-}$ and *Ahr*$^{-/-}$ Mice Exposed to Cannabis Smoke vs Air

(A) Venn diagram displaying the total number of DEPs in *Ahr*$^{+/-}$ and *Ahr*$^{-/-}$ mice exposed to cannabis smoke compared to air-exposed controls. (B) Bar graph quantifying the number of DEPs that are upregulated or downregulated in each genotype. (C, D) Volcano plots illustrating the distribution of DEPs in (C) *Ahr*$^{+/-}$ and (D) *Ahr*$^{-/-}$ mice. The horizontal dotted line indicates the statistical significance threshold (p<0.05).

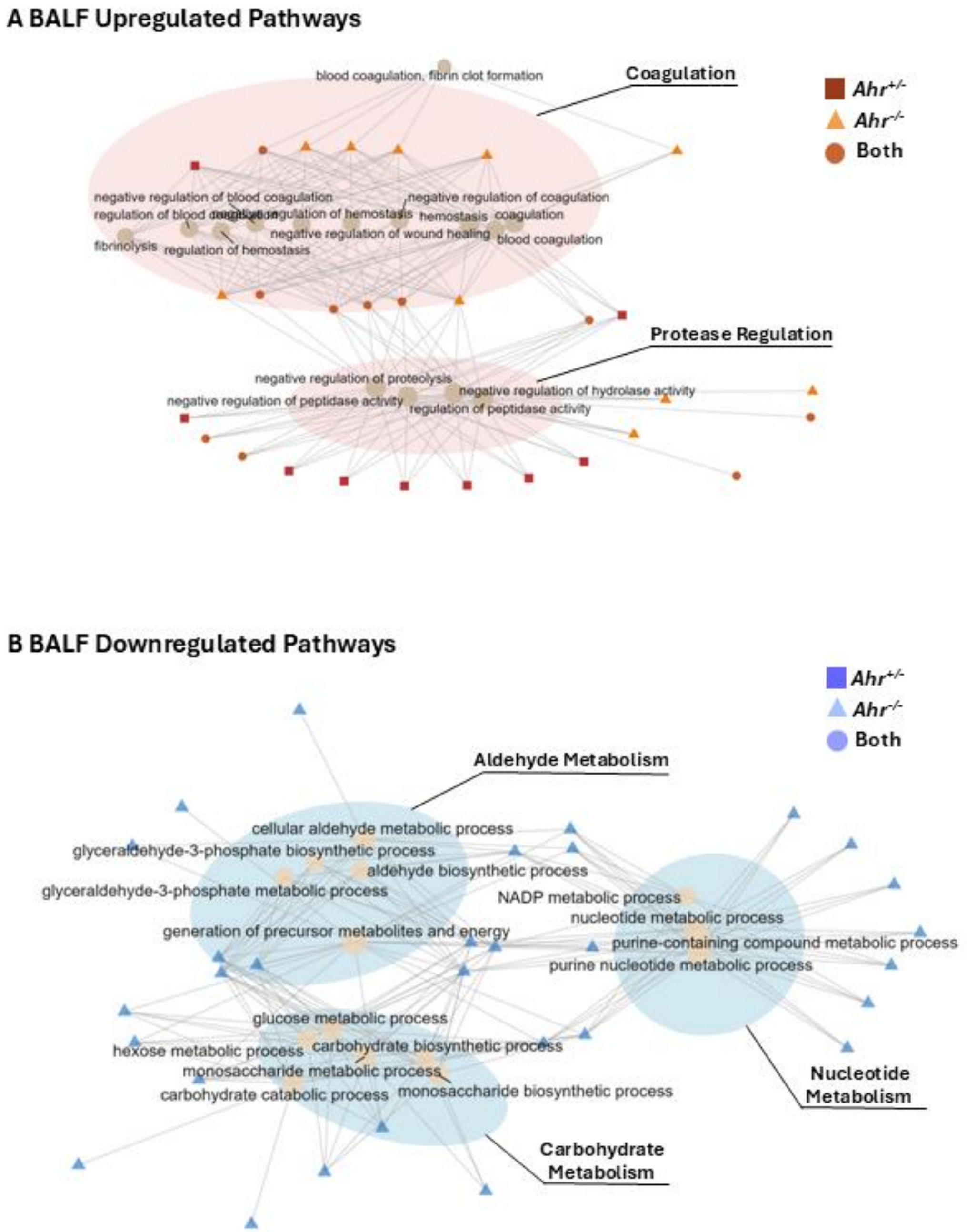

Supp. Figure 11 Labelled Network Analysis of Top 15 Upregulated and Downregulated Pathways in BALF.

(A) Network analysis of the top 15 upregulated pathways in BALF following cannabis smoke exposure. (B) Network analysis of the top 15 downregulated pathways in EVs following cannabis smoke exposure. Beige pathway nodes are labeled with the GO term they represent, while the protein nodes are color-coded based on the genotype in which they are differentially expressed.

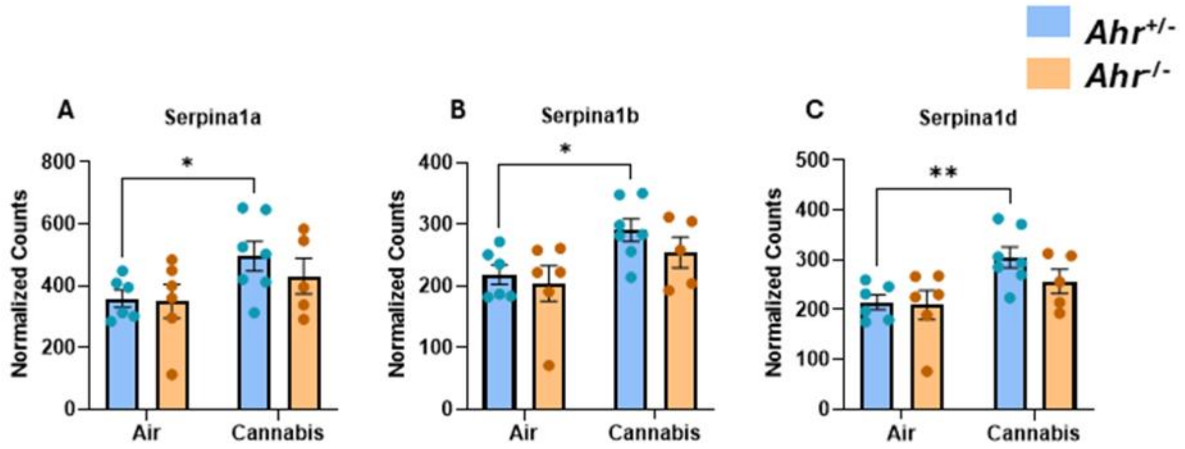

Supp. Figure 12 Levels of A1AT Variants in BALF of *Ahr*$^{+/-}$ and *Ahr*$^{-/-}$ Mice Exposed to Air or Cannabis Smoke.

Normalized counts of (A) Serpina1a, (B) Serpina1b, and (C) Serpina1d in BALF. Data are presented as mean ± SEM, with individual data points shown. Statistical significance is denoted by *p < 0.05, and **p < 0.01.
